# Rare Gallbladder Carcinoma: A Case Report on Squamous Cell Carcinoma of the Gallbladder

**DOI:** 10.1155/cris/8849920

**Published:** 2026-11-04

**Authors:** Cassandra Nathan, Benjamin H. Russell, Joseph Sferra

**Affiliations:** ^1^ Department of General Surgery University of Toledo Toledo Ohio USA utoledo.edu; ^2^ Department of Surgery Promedica Toledo Hospital and Health Systems Toledo Hospital Toledo Ohio USA toledoresidency.org

**Keywords:** case report, gallbladder carcinoma, genetic testing, squamous cell carcinoma

## Abstract

Squamous cell carcinoma (SCC) of the gallbladder is a rare and aggressive subtype that typically presents at an advanced stage and carries a poor prognosis, often with median survival under 1 year. Risk factors reflect those of other gallbladder cancers—chronic cholecystitis, gallstones, and female sex. Diagnosis is frequently made intra‐ or postoperatively via histopathological biopsy. Surgical resection is the primary treatment, but curative intervention is often unachievable due to extensive disease at presentation. We describe a 59‐year‐old woman with fevers, aches, chills, night sweats, nausea, and vomiting with imaging suggestive of a liver abscess with fistulous connection to the gallbladder. Exploratory laparotomy revealed poorly differentiated, nonkeratinizing SCC with sarcomatoid features; immunohistochemistry was positive for CK5/6, p40, and CK7. Understanding the molecular mechanisms for the progression of SCC of the gallbladder could help target interventions. Given its rarity, standardized adjuvant treatment guidelines are lacking—highlighting the need for further molecular and therapeutic research.

## 1. Introduction

Gallbladder cancer is the most common malignancy of the biliary tract, with adenocarcinoma comprising 80%–90% of cases [1]. Squamous cell carcinoma (SCC) of the gallbladder is a very rare form of gallbladder cancer with a poor prognosis [2]. It accounts for 1%–4% of all gallbladder carcinoma (GBC) cases and is more aggressive when compared to adenocarcinoma of the gallbladder, with a median survival of 7 months after diagnosis [2]. Due to its rarity, there is limited data in the literature. Existing literature, including case reports and small case series, suggests that SCC is more locally invasive than adenocarcinoma [1, 3].

Risk factors for SCC are the same as all GBCs and include older age, female sex, chronic cholecystitis, cholelithiasis, gallbladder polyps, biliary Salmonella typhi infection, chronic biliary inflammation, family history, and aflatoxin exposure [4].

SCC is typically diagnosed intraoperatively via biopsy and at a later stage and histological grade [2, 5]. An analysis of the National Cancer Database by Ayabe et al. [1] reports that almost half (40%) of gallbladder SCC (GBSCC) present with stage IV disease, with the liver being the most reported site of metastasis. Many of the previous case reports noted concomitant cholelithiasis or cholecystitis [6].

Standard treatment includes complete surgical resection. However, due to the aggressiveness of the cancer and its later presentation, it may be difficult to achieve clear R0 margins. Even when full resection of the tumor is achieved, SCC of the gallbladder has worse survival rates compared to adenocarcinoma [1, 7]. There is some evidence that adjuvant radiation therapy after full surgical resection with negative margins may provide favorable outcomes for SCC of the gallbladder [8].

Here, we present a case of a 59‐year‐old female with concern for hepatic abscess secondary to a fistulous connection between the gallbladder and the liver that turned out to be metastasis from SCC of the gallbladder. To the best of our knowledge, this is the fifth case of SCC of the gallbladder initially presenting as a liver abscess.

## 2. Case Presentation

A 59‐year‐old female with a medical history of diabetes mellitus type II and hypothyroidism presented to the emergency room after 5 weeks of fever, achiness, night sweats, nausea, and vomiting. At presentation, she had a white blood cell count (WBC) of 29,000 cells/μL and a urine culture growing *E. coli*. Physical exam was pertinent for tenderness in the right upper quadrant of the abdomen without focal peritonitis. A CT abdomen and pelvis without contrast was performed (Figure 1, blue arrow), showing multiple hypoattenuating lesions within the liver, with concern for hepatic abscess versus hepatic metastatic disease, and equivocal cholecystoduodenal fistula. A dominant hypoattenuating lesion within hepatic segment IVb/V containing a large peripherally calcified lesion was also noted (Figure 2a,b, blue arrow).

**Figure 1:**
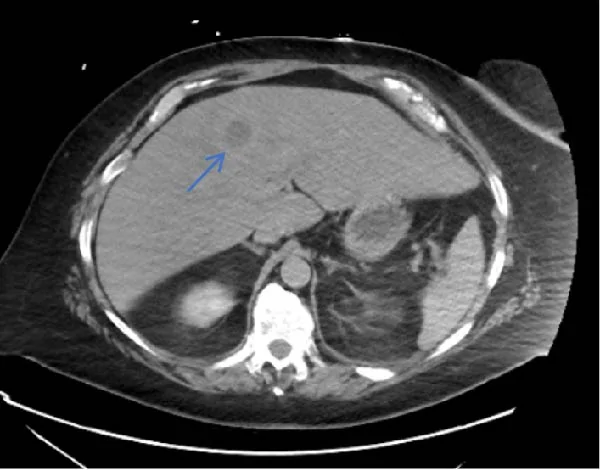
CT abdomen pelvis without contrast, axial view showing hepatic lesions (blue arrow).

**Figure 2:**
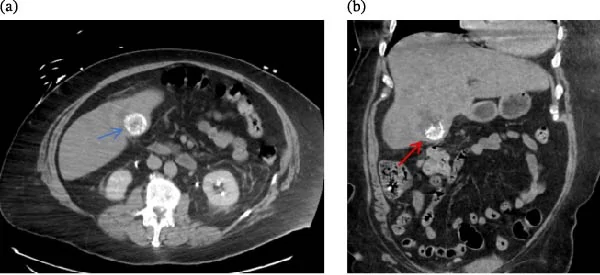
(a) Axial CT abdomen/pelvis showing hepatic segment IVb/V containing a large peripherally calcified lesion (blue arrow). (b) Coronal CT abdomen/pelvis showing hepatic segment IVb/V containing a large peripherally calcified lesion (red arrow).

Due to the hepatic abscesses and a cholecystoduodenal fistula noted on CT, an MRCP was ordered for further characterization of the biliary tree and liver. The MRCP showed findings most consistent with multifocal hepatic abscesses throughout segment IV, a fistulous connection between the gallbladder and hepatic parenchyma, and cholelithiasis (Figure 3, red arrow). The multifocal abscesses were noted to be stemming from the gallbladder, thus favoring hepatic abscess over the diagnosis of malignancy. An open cholecystectomy with liver abscess drainage was recommended. Oncology was following and agreed with the plan for drainage of liver abscesses to obtain tissue biopsy, as initial suspicion for malignancy was low. However, tumor markers were sent and notable for carcinoembryonic antigen (CEA) of 6.2 ng/mL, alpha‐fetoprotein (AFP) of 1.4 ng/mL, cancer antigen (CA) 19‐9 of 33.6 U/mL, and CA 125 of 96 U/mL.

**Figure 3:**
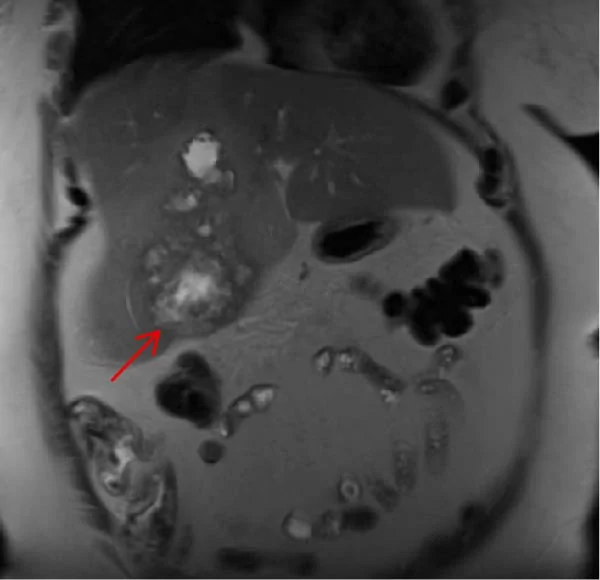
T2 Haste MRCP showing fistulous connection between the gallbladder and hepatic parenchyma and cholelithiasis (red arrow).

Intraoperatively, the liver was found to have multiple masses on its surface, suspicious for metastatic disease, and biopsies were taken. Upon continued exploration, the gallbladder appeared chronically inflamed and densely adherent to its surroundings. The anterior wall of the gallbladder was found to be fused with the omentum, and upon separation, an abscess cavity was discovered. Purulent and bilious material was evacuated and sent for microbiology. The gallbladder was entered, and a large tumor within the gallbladder lumen was encountered. The tumor had also perforated through the posterior gallbladder wall into the liver parenchyma. Numerous gallstones were also found. A subtotal fenestrating cholecystectomy was performed due to significant dense adhesions between the posterior gallbladder wall and the liver parenchyma. Intraoperative specimens sent for frozen sections were consistent with SCC. A hematoxylin and eosin (H&E) stain under 10× magnification (Figure 4) demonstrated nests and sheets of SCC cells dissecting the smooth muscle bundles of the gallbladder. A 40× magnification H&E stain (Figure 5, white arrow) demonstrated an invasive SCC forming a keratinized pearl with adjacent lymphoid infiltrate. Intraoperative ultrasonography was performed, which showed numerous additional masses within the liver parenchyma. After irrigating the abdomen and introducing a drain into the gallbladder fossa, the abdomen was closed.

**Figure 4:**
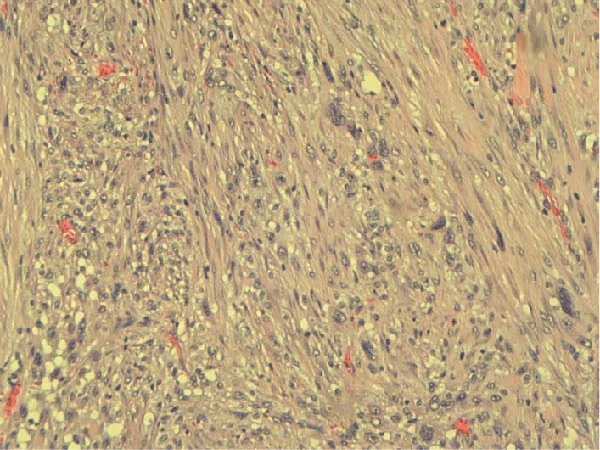
Nests and sheets of squamous cell carcinoma cells dissecting the smooth muscle bundles of the gallbladder (H&E stain; 10× magnification).

**Figure 5:**
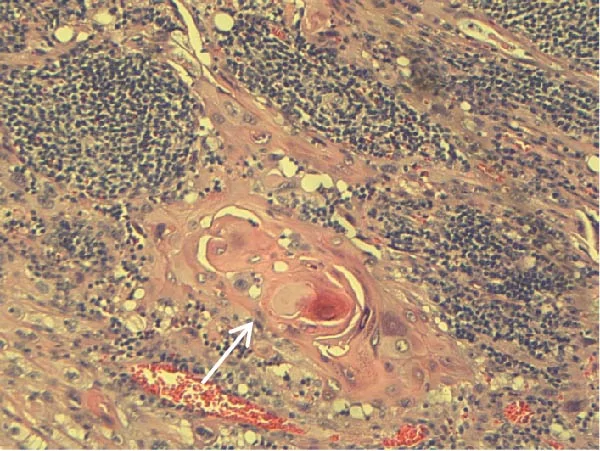
Invasive squamous cell carcinoma forming a keratinized peal with adjacent lymphoid infiltrate (H&E stain; 40× magnification) (white arrow).

Pathology of the liver biopsy revealed metastatic SCC with sarcomatoid features, poorly differentiated, and nonkeratinizing. The gallbladder specimen showed carcinoma involving most of the gallbladder with direct invasion into the liver. Metastatic carcinoma was noted in 2 of 2 lymph nodes. The gallbladder specimen stained positive for CK5/6, p40, and CK7 and was negative for CK20 and CDX2. CK 5/6 and p40 are markers of squamous cell differentiation; expression in a tissue sample from the gallbladder suggests squamous or adenosquamous carcinoma, as they are infrequently positive in gallbladder adenocarcinoma. CK7 expression is seen in the gallbladder mucosa, representing epithelial differentiation. CK20 is normally expressed in the gastrointestinal tract; thus, a CK7+/CK20‐ phenotype is more consistent with pancreaticobiliary instead of a metastasis from a colorectal primary. CDX2 is a transcription factor in the intestinal epithelium, and positivity in a gallbladder specimen indicates intestinal metaplasia or differentiation.

Due to the metastatic nature at presentation, a complete resection was not possible. Postoperatively, the patient was discharged home and set to follow up with hematology/oncology on an outpatient basis. A month after surgery, she developed poor functional status with multiple falls and sepsis, prompting repeat admissions in the hospital. Physical therapy and occupational therapy had recommended a skilled nursing facility versus inpatient rehab; however, she refused both, stating that she wanted to recover at home. At her final admission 2 months after index operation, she was deemed not a candidate for systemic chemotherapy due to poor functional status and newly developed nutritional deficiencies. She continued to decline and passed away 2 months after diagnosis. Unfortunately, due to her rapid decline, we were unable to obtain her perspective on treatment and management.

## 3. Discussion

GBC is one of the most aggressive types of malignancies of the gastrointestinal tract and often found at later stages due to its potential to metastasize [9]. Of all the subtypes of GBC, SCC is the rarest. Currently, there is limited data on the epidemiology and management of this tumor. As seen in our case, most patients are older females who present with general abdominal pain, nausea, and fever and are found to have concurrent cholecystitis [10].

There are a few different hypotheses for the etiology of GBSCC due to the fact that there is no squamous epithelium in the gallbladder [11]. One suggestion is that ectopic squamous epithelium undergoes malignant transformation [12]. Another describes the process of metaplastic squamous epithelium progressing to dysplasia and, ultimately, to malignant tissue. Chronic irritation of the gallbladder from gallstones may trigger differentiation of glandular cells into squamous cells [13]. A third hypothesis suggests that mixed adenosquamous carcinoma of the gallbladder eventually undergoes excessive growth of the squamous cells and ultimately replaces the adenocarcinoma component [14].

On surgical pathology, our patient’s tumor was reported to consist of fragments of invasive SCC with sarcomatoid features and keratinization, which is similar to other prior case reports of GBSCC [12]. Immunohistochemical staining of our patient’s tumor was positive for CK5/6, p40, and CK7 and negative for CK20 and CDX2. CK7 positivity along with CK20 negativity has been reported in almost half of all GBCs, especially when presenting with concurrent cholelithiasis [15].

When discussing the treatment of GBSCC, full R0 resection provides the best prognosis [2, 4]. However, because our patient’s tumor had already invaded the liver, a full resection was not possible. Aside from surgical resection, treatment consists of both systemic chemotherapy and radiation; however, prognosis is poor [15]. Current National Comprehensive Cancer Network (NCCN) guidelines recommend gemcitabine plus cisplatin with or without immune checkpoint inhibitors (durvalumab or pembrolizumab).

Understanding the molecular mechanisms for the progression of SCC of the gallbladder could help identify a target for future interventions. While the data are limited, a few studies have shown that there may be an association between CD109 and the development of GBSCC. CD109 is a coreceptor for TGFB1 and suppresses TGF‐B signaling, which subsequently promotes SCC progression [16]. CD109 has also been found to be a regulator of EGFR expression at the mRNA and protein level [17]. A recent study showed that the CD109‐EGFR complex maintains epithelial morphology and shows in vitro tumorigenicity [17]. Studies have shown that CD109 is expressed in pure SCCs and adenosquamous carcinomas but not in adenocarcinomas or normal gallbladder tissues [18]. These results demonstrate that CD109 and EGFR may be involved in the pathogenesis of GBSCC and could be a target for molecular therapy in the treatment of SCC. Due to the rarity and aggressive behavior of SCC of the gallbladder and limited case reports, the immunohistochemical stains that are positive in our case may not be generally applicable to all cases of SCC of the gallbladder.

SCC of the gallbladder is a rare aggressive subtype of gallbladder cancer. Current management is surgical resection combined with chemotherapy with or without immune checkpoint inhibitors. We present a case of SCC of the gallbladder with immunohistochemical staining that may identify new targets for molecular therapy.

## Funding

The authors received no funding for the publication of this article.

## Ethics Statement

Institutional Review Board approval was not required for this single anonymized case report.

## Consent

Informed written consent was obtained for the inclusion of anonymized patient information in the publication of this case report.

## Conflicts of Interest

The authors declare no conflicts of interest.

## Supporting Information

Additional supporting information can be found online in the Supporting Information section.

## Supporting information


**Supporting Information** CARE Checklist of information to include when writing a case report.

## Data Availability

The data that support the findings of this study are available from the corresponding author upon reasonable request.
